# Efficiency improvement of spin-resolved ARPES experiments using Gaussian process regression

**DOI:** 10.1038/s41598-024-66704-8

**Published:** 2024-09-23

**Authors:** Hideaki Iwasawa, Tetsuro Ueno, Takuma Iwata, Kenta Kuroda, Konstantin A. Kokh, Oleg E. Tereshchenko, Koji Miyamoto, Akio Kimura, Taichi Okuda

**Affiliations:** 1 Synchrotron Radiation Research Center, National Institutes for Quantum Science and Technology, Sayo, 679-5148 Japan; 2 NanoTerasu Center, National Institutes for Quantum Science and Technology, Sendai, 980-8579 Japan; 3 Research Institute for Synchrotron Radiation Science, Hiroshima University, Higashi-Hiroshima, 739-0046 Japan; 4 Quantum Materials and Applications Research Center, National Institutes for Quantum Science and Technology, Takasaki, 980-8579 Japan; 5 Graduate School of Advanced Science and Engineering, Hiroshima University, Higashi-Hiroshima, 739-8526 Japan; 6 International Institute for Sustainability with Knotted Chiral Meta Matter (WPI-SKCM2), Hiroshima University, Higashi-Hiroshima, 739-8526 Japan; 7 V. S. Sobolev Institute of Geology and Mineralogy, Siberian Branch, Russian Academy of Sciences, Novosibirsk, 630090 Russia; 8 Saint Petersburg State University, Saint Petersburg, 198504 Russia; 9 Rzhanov Institute of Semiconductor Physics, Siberian Branch, Russian Academy of Sciences, Novosibirsk, 630090 Russia; 10 Synchrotron Radiation Facility SKIF, Boreskov Institute of Catalysis, Siberian Branch, Russian Academy of Sciences, Kol’tsovo, 630559 Russia; 11 Research Institute for Semiconductor Engineering (RISE), Hiroshima University, Higashi-Hiroshima, 739-8527 Japan

**Keywords:** Techniques and instrumentation, Condensed-matter physics

## Abstract

The experimental efficiency has been a central concern for time-consuming experiments. Spin- and angle-resolved photoemission spectroscopy (spin-resolved ARPES) is renowned for its inefficiency in spin-detection, despite its outstanding capability to directly determine the spin-polarized electronic properties of materials. Here, we investigate the potential enhancement of the efficiency of spin-resolved ARPES experiments through the integration of measurement informatics. We focus on a representative topological insulator $$\text {Bi}_{2}$$$$\text {Te}_{3}$$, which has well-understood spin-polarized electronic states. We employ Gaussian process regression (GPR) to assess the accumulation of spin polarization information using an indicator known as the GPR score. Our analyses based on the GPR model suggest that the GPR score can serve as a stopping criterion for spin-resolved ARPES experiments. This criterion enables us to conduct efficient spin-resolved ARPES experiments, significantly reducing the time costs by 5-10 times, compared to empirical stopping criteria.

## Introduction

The spin polarization and spin texture of electronic states are crucial for understanding the physical properties of materials, particularly in fields such as spintronics and quantum materials. Therefore, spin-resolved and angle-resolved photoemission spectroscopy (spin-resolved ARPES) has been at the forefront of research in these fields in recent years, thanks to its ability to provide valuable information about the spin-polarized electronic properties of materials^1,2^. Although the spin-resolved ARPES experiment has been plagued by inherent inefficiencies in spin detection, the efficiency has been consecutively and revolutionarily improved nowadays. The efficiency of the spin-resolved ARPES experiment is determined by the spin sensitivity of the spin detector and electron scattering probabilities. The spin sensitivity is commonly known as the effective Sherman function ($S_\text {eff}$), while the electron scattering probabilities by the target are represented by the ratio $I/I_{0}$, where *I* denotes the total intensity of the beam measured in the two scattering channels, and $I_0$ represents the intensity of the incident electron beam. The overall efficiency of the spin-resolved ARPES experiment using a single channel spin detector is then expressed by the figure-of-merit, ${\mathscr {F}}_{1D} = S_\text {eff}^2 I/I_{0}$.

There are two major types of spin detectors based on spin-orbit and spin-exchange interactions, having different characteristics. The Mott detector, which is the most commonly used spin detector based on the spin-orbit interaction^3^, typically provides an ${S_{\text {eff}}}$ ranging from 0.1 to 0.2 and a scattering probability on the order of $10^{-2}$. The resulting ${{\mathscr {F}}_{1D}}$ is thus significantly low, on the order of $10^{-4}$, requiring $10^4$ times longer acquisition time to get a spectrum with an equivalent signal-to-noise (*S*/*N*) ratio as conventional spin-*integrated* ARPES experiments. Alternative methods have been developed based on the spin-exchange interaction, the so-called very low energy electron diffraction (VLEED) spin detector^4^. The VLEED spin detector utilizes the asymmetry in the reflected intensity between photoemitted electrons and magnetically ordered surfaces of target materials at energies below 10 eV. The scattering intensity is significantly strong, resulting in ${{\mathscr {F}}_{1D}} {\sim } 10^{-2}$, nearly 100 times improved efficiency compared to Mott-type spin detectors, under the assumption of no intensity fluctuations in the incident light^5^. Accordingly, high-resolution spin-resolved ARPES experiments have become feasible thanks to the improved efficiency provided by the VLEED spin detector^1,6^. Very recently, further progress in efficiency has been achieved by developing an imaging-type multichannel spin detector. The effective figure-of-merit increases with the number of detection channels *N*, as ${{\mathscr {F}}_{2D}} = N \times {{\mathscr {F}}_{1D}}$. Typically, imaging-type multichannel spin detectors can measure $N=10^{3}-10^{4}$ points simultaneously, resulting in remarkably high ${\mathscr {F}}_{2D}$ values ranging from 10 to $10^2$ of orders. So far, several imaging-type multichannel spin detectors have been developed, including a VLEED-based multichannel spin detector^7^, an imaging spin-filter in a momentum microscope (MM)^8^, and a time-of-flight MM^9^. Accordingly, the efficiency of spin-resolved ARPES experiments has dramatically improved through the development of innovative spin detectors.

On the other hand, measurement informatics has recently gained significant attention for its ability to enhance experimental efficiency^10–13^. There are two primary streams in measurement informatics: one focuses on data analysis, while the other is concerned with data acquisition, often referred to as ‘measurement’ in a narrow sense. The application of informatics for data analysis serves various purposes, including efficient and reproducible analysis of extensive datasets, eliminating preconceptions of human analysts, and freeing them from monotonous repetitive tasks. In spectroscopic experiments, for instances, automated peak fitting with Bayesian optimization^14,15^or expectation-conditional maximization (ECM) algorithm^16^, and clustering of spatially-resolved ARPES spectra have been reported^17,18^. Moreover, feature extraction of electron energy-loss near-edge structure (ELNES) and X-ray absorption near-edge structure (XANES) spectra have been reported^19,20^. For diffraction experiments, there are reports, such as classification of crystal system and space groups from X-ray diffraction (XRD) data by interpretable^21^ or deep-learning approach^22^, Bayesian inference in crystallographic structure refinement^23^, automated Rietveld analysis by black-box optimization^24^, and the extraction of ‘materials concepts’ from XRD database^25^.

Application of informatics to data acquisition is aimed for reducing measurement time and cost, which is especially crucial in large-scale experimental facilities like synchrotron-radiation or neutron facilities, where the allocation of machine time for individual users is limited. Moreover, experimental throughput increases not only in large-scale facilities but also in small laboratories, thanks to aforementioned methodologies. Therefore, improving experimental efficiency through measurement informatics is a practical approach to overcome such situations. One approach for reducing measurement time is reducing the number of measurement data points. For X-ray absorption and X-ray magnetic circular dichroism spectroscopy, active learning by Gaussian process regression (GPR) successfully reduced measurement data points^26,27^. Another is reducing the integration time for one measurement, particularly applicable for scattering experiments using 2D detectors. Kernel density estimation (KDE) was applied to small-angle X-ray scattering (SAXS)^28^ and small-angle neutron scattering (SANS)^29^ experiments, proving helpful in reducing measurement time. In addition, the automated stopping of experiment without intervention of human experimenters is an essential but on-going challenge^27^. Furthermore, measurement informatics is expected to be integrated with autonomous experimental systems reinforced by robotics technologies to improve the efficiency of the entire system^30–34^. Consequently, the integration of measurement informatics into spectroscopic measurements is becoming more common, aiming to enhance experimental design and efficiency while minimizing arbitrariness and reducing the workload associated with human intervention.

In this work, we aim to investigate the possibility to improve the efficiency of the spin-resolved ARPES experiments through the utilization of the measurement informatics. To eliminate any potential uncertainty, we selected $$\text {Bi}_{2}$$$$\text {Te}_{3}$$, a representative topological insulator that exhibits the clear spin-polarization and its spin-polarized electronic states are well-understood^2,35^. We employed a GPR model to assess how the information on spin polarization is accumulated, and to explore suitable indicators for the spin-resolved ARPES experiments. Our analyses and discussions based on the GPR model demonstrate that the accumulation of spin polarization information can be described by an indicator known as the GPR score, which can serve as a stopping criterion for spin-resolved ARPES experiments. This criterion enables us to perform efficient spin-resolved ARPES experiments, significantly reducing the time cost approximately 5-10 times lower than that obtained using an empirical stopping criterion.

## Results and discussion

**Figure 1:**
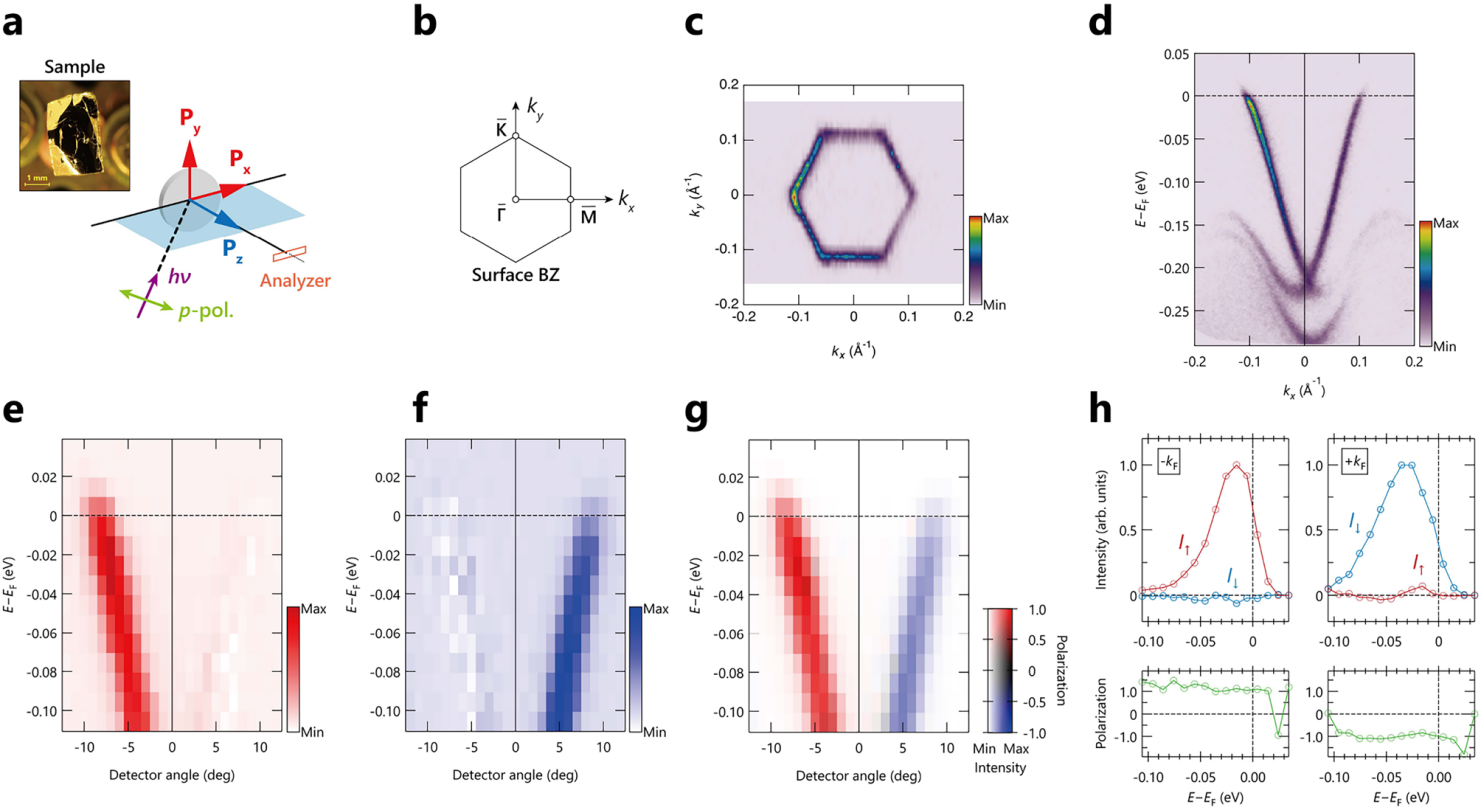
Overview of the spin-resolved ARPES experiments on a topological insulator $$\text {Bi}_2$$$$\text {Te}_3$$, including the experimental configuration and representatives of ARPES and spin-resolved ARPES results. (**a**) Experimental configuration of the present spin-resolved ARPES measurements as well as optical microscope image from the cleaved $$text{Bi}_2$$ $$\text {Te}_3$$ surface taken *ex-situ* after experiments. The polarization direction of incident light is parallel (*p*-polarization) to the analyzer slit unless specified. (**b**) Schematic view of the surface Brillouin zone of hexagonal $$\text {Bi}_2$$$$\text {Te}_3$$. (**c** and **d**) Spin-integrated ARPES Fermi surface and high-symmetry image along the $${\overline{\Gamma }}-\overline{\text {M}}$$ line. (**e**–**g**) Spin-resolved ARPES image along the $${\overline{\Gamma }}-\overline{\text {M}}$$ line for spin-up (**e**) and spin-down (**f**) states, and corresponding spin-polarization map (**g**). (**h**) Spin-resolved EDCs and spin polarization, respectively, at Fermi momenta ($${\pm k_{\text {F}}}$$) along the $${\overline{\Gamma }}-\overline{\text {M}}$$ line.

Figure 1 provides an overview of the present spin-resolved ARPES experiments and dataset on $$\text {Bi}_2$$ $$\text {Te}_3$$. The experimental configuration is schematically depicted in Fig. 1a, along with the optical microscope image of the cleaved surface of the sample. The laser light is incident on the sample at a $$50^{\circ }$$ from the focal axis of the electron analyzer. Unless specified otherwise, we employed the *p*-polarization, meaning the vector potential is in-plane with the photoelectron detection plane and parallel to the horizontal slit of the electron analyzer. With this experimental setup, we examined the in-plane component of spin polarization $$P_{y}$$, tangential to the hexagonal Fermi surface of $$\text {Bi}_2$$$$\text {Te}_3$$, as illustrated in Fig. 1a,b. Before conducting the spin-resolved ARPES experiments, we measured spin-integrated ARPES data of the Fermi surface and ARPES image along the high-symmetry $${\overline{\Gamma }}-\overline{\text {M}}$$ line, as shown in Fig. 1c,d, respectively, demonstrating high-quality data essential for the objectives of this work.

$$\text {Bi}_2$$$$\text {Te}_3$$ is known for a three-dimensional topological insulator, which exhibit metallic surface states consisting of a single Dirac cone at the $${\overline{\Gamma }}$$ point. Given the expected spin-polarized electronic states with a helical spin-texture for $$P_{y}$$, either spin-up or spin-down states are observed at positive and negative Fermi momenta ($$\pm k_{\text {F}}$$), symmetric with respect to the $${\overline{\Gamma }}$$ point, as shown in Fig. 1e,f, respectively. The reversal of the spin polarized direction can be more clearly visualized in the spin-polarization map in Fig. 1g. In Fig. 1h, we show the spin-resolved energy distribution curves (EDCs), where the spin-up and spin-down signals are indicated by the red and blue curves, respectively, accompanied by the corresponding spin-polarization shown in green curves. One can easily see the clear and almost completely polarized (100$$\%$$) spin-up and spin-down states at $$\pm k_{\text {F}}$$. In this work, we utilized the spin-up states as a showcase for the application of measurement informatics in spin-resolved ARPES experiments.

**Figure 2:**
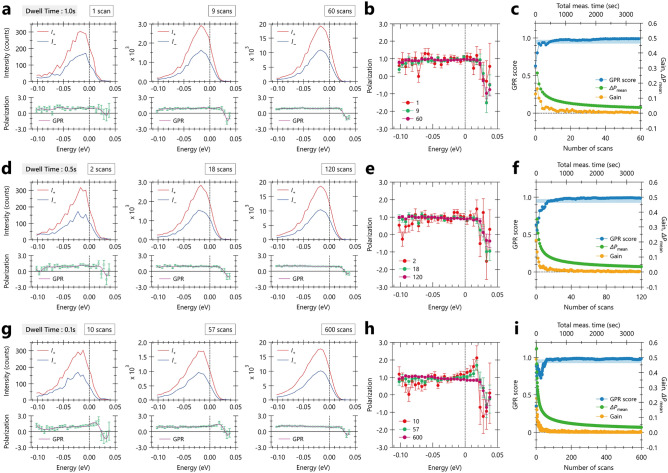
Evaluation of accuracy in spin-polarization extracted from the spin-resolved EDCs with different dwell times and accumulations in $$\text {Bi}_{2}$$$$\text {Te}_{3}$$ using Gaussian process regression (GPR) model. (**a**,**d**,**g**) Raw EDCs for positive (red) and negative magnetization (blue), along with the spin-polarization (green), for different dwell times ($$T_\text {dwell}$$’s: 1.0, 0.5, and 0.1 sec.) and several numbers of scans ($$N_\text {scan}$$’s). Here, the first, critical ($$N_\text {cr}$$), and last number of scans ($$N_\text {emp}$$) are highlighted for $$N_\text {scan}$$’s. The definitions of $$N_\text {cr}$$ and $$N_\text {emp}$$ are given in the main text. The mean of GPR prediction (purple) is overlaid on the experimental spin-polarization, while the standard deviation of GPR prediction is also plotted but too small to be visible by the eye. (**b**,**e**,**f**) Comparison of spin-polarization, including uncertainty, between different $$N_\text {scan}$$’s for different $$T_\text {dwell}$$’s. (**c**,**f**,**i**) GPR score (light blue) on the left axis, as well as gain and mean uncertainty of the spin-polarization $${\Delta }P_{\text {mean}}$$ (orange and green curves) on the right axis, as a function of $$N_\text {scan}$$, for different $$T_\text {dwell}$$’s (bottom axis) and total measurement times (top axis), given by $$2 \times T_\text {dwell} \times n \times N_\text {scan}$$, where *n* represents the number of energy points.

One of the primary objectives of spin-resolved ARPES experiments is to extract information about the spin polarization of materials. In our spin-resolved ARPES experimental setup utilizing the VLEED-type spin detector, the spin polarization (*P*) is determined as the asymmetry between spin-up ($I_{\uparrow }$) and spin-down states ($I_{\downarrow }$). This asymmetry is proportional to the difference in the scattered electron intensity between positively and negatively magnetized targets ($I_{+}$ and $I_{-}$) in the VLEED detector. The spin-polarization can then be determined using the formula $P=S^{-1}(I_{+}-I_{-}) / (I_{+}+I_{-})$, with the Sherman function $S=0.3$ in our present system^36^. More specifically, in this work, we measured the spin-resolved EDCs. This involves measuring pairs of $I_{+}$ and $I_{-}$ as a function of energy (*E*), namely, $I_{+}(E)$ and $I_{-}(E)$, where $E = (E_1, \ldots ,E_n)$ with the number of energy points (*n*), with a certain dwell time ($T_\text {dwell}$) and a number of scans ($N_\text {scan}$). Consequently, the time-cost for obtaining a spin-polarization *P* is essentially given by $2 \times T_\text {dwell} \times n \times N_\text {scan}$, excluding any potential waiting time and/or dead-time associated with practical measurements. While lower $T_\text {dwell}$ and $N_\text {scan}$ is preferable from the viewpoint of experimental efficiency, the accuracy of the spin-polarization (${\Delta }{P}$) is, of course, a critical parameter in spin-resolved ARPES experiments, where ${\Delta }{P}$ is given by $S^{-1} I^{-1/2}$ using the total intensity $I = I_{+}+I_{-}$. Therefore, the efficiency of spin-resolved ARPES experiments has to be optimized while considering the overall balance among these parameters ($T_\text {dwell}$, $N_\text {scan}$, and ${\Delta }{P}$). One practical approach for achieving efficient spin-resolved ARPES experiments is to repeat measurements with short $T_\text {dwell}$ while evaluating the accuracy of the spin polarization. In general, the accuracy should increase while accumulating $N_\text {scan}$, being a possible stopping criteria of experiments. However, it is challenging issue to generalize, as it depends on the objective parameters and type of experimental dataset, which rely on experimental methods. Here, we explore a stopping criterion for spin-resolved ARPES experiments utilizing the GPR model against two types of dataset, consist of different $T_\text {dwell}$’s and magnitudes of $P_y$. We will discuss these aspects below in order.

### ${{\varvec{T}}}_{{{\textbf {dwell}}}}$ dependence

Figure 2 shows the evaluation of accuracy of the spin-polarization in spin-resolved ARPES experiments on $$\text {Bi}_2$$$$\text {Te}_3$$, taken with different dwell times ($$T_\text {dwell}$$’s: 1.0, 0.5, and 0.1 sec.) as a function of numbers of scans ($$N_\text {scan}$$’s). For these $$T_\text {dwell}$$’s, the spin-polarization (green) was extracted from raw spin-resolved EDCs for positive (red) and negative magnetization (blue), as shown in Fig. 2a,d,g. We further approximated the obtained spin-polarization by the GPR model (for details, see Methods), to examine how the information on the spin-polarization accumulates with increasing $$N_\text {scan}$$. The resulting mean of GPR prediction, along with its standard deviation, is overlaid on the spin-polarization, although the standard deviation is too small to be visible by the eye. Note that the accumulation of the *S*/*N* ratio of the spin-polarization is more clearly seen in Fig. 2b,e,h, where one can notice the large uncertainty in the spin-polarization for higher energy. However, this uncertainty should be ignored because of the almost zero intensities of $$I_{+}$$ and $$I_{-}$$, which is a natural consequence of $$E > E_\text {F}$$.

Then, the goodness of the fit between the experimental data and the GPR prediction can be evaluated by the GPR score, as shown on the left axis in the Fig. 2c,f,i, along with the information gain of measurement *G* and the mean uncertainty of the spin polarization ${\Delta }{P}$ on the right axis. Here, we defined *G* as $G = \sum |\mu _{i-1} - \mu _{i}|/\sum \mu _{i}$, where $\mu _{i}$ is the predicted mean of GPR for the present scan and $\mu _{i-1}$ is that of the previous one (see also Methods). We found that the GPR score increases rapidly and then becomes almost saturated nearby one, while *G* and ${\Delta }{P}$ show the opposite trend, namely, decreasing rapidly and then saturating. We then assume the critical number of scans ($N_\text {cr}$), at which the GPR score reaches 95$\%$ or higher, meaning that the information on the spin-polarization is adequately accumulated. The $N_\text {cr}$ is found to be 9, 18, and 57 scans for $T_\text {dwell}$’s of 1.0, 0.5, and 0.1 sec., respectively. Surprisingly, these values are much smaller than the last number of scans determined in an empirical manner ($N_\text {emp}$). The $N_\text {emp}$ is typically determined to satisfy $I_{\pm }^\text {max} > 10^4$, and $N_\text {emp}$ was 60, 120, and 600 for $T_\text {dwell}$’s of 1.0, 0.5, and 0.1 sec, respectively. It should also be noted that the ${\Delta }{P}$ becomes saturated towards ${\Delta }{P} = 2.4~\%$ with $S = 0.3$ and $I = 2 \times 10^4$. Accordingly, we can improve the efficiency of spin-resolved ARPES experiments by $N_\text {emp} / N_\text {cr}$, as 6.7, 6.7, and 10.5 for $T_\text {dwell}$’s of 1.0, 0.5, and 0.1 sec, respectively, by reasonably reducing the acquisition number of scans. The reasonable reduction of the acquisition number of scans can also be supported by the almost similar appearance of the obtained raw EDCs and spin-polarization at $N_\text {cr}$ and $N_\text {emp}$. Therefore, the present GPR model analysis, utilizing the GPR score as the stopping criteria, enabled 5-10 times improvement in efficiency in the present case. However, it should be emphasized here that it is essential to consider the balance between the magnitude of the spin-polarization and its accuracy − in this case, they are ${\sim }100\%$ and ${\sim }10\%$ at $N_\text {cr}$, respectively. In other words, it has to be taken into account whether the accuracy is enough to validate the obtained magnitude of the spin-polarization in spin-resolved ARPES experiments.

On the other hand, it may be noticeable that the GPR score does not monotonically increase for $T_{\text {dwell}} = 0.1$, as seen in the dip at $N_\text {scan} \sim 25$. This suggests that the *S*/*N* ratio is not simply accumulated in the case of the short $T_{\text {dwell}}$, implying higher counts of noise ($I_\text {noise}$) compared to signal ($I_\text {signal}$), where we assume the total counts $I_\text {total} = I_\text {signal} + I_\text {noise}$. There are two plausible explanations for this. One explanation is that the time allowing for stable counting of the signal might be shorter or comparable to $T_{\text {dwell}}$. This time should be specific depending on detection systems, and we define it as the optimum measurement time, $T_{\text {dwell}}^{\text {opt}}$. It is reasonable that intensity fluctuations occur in $I_\text {signal}$ when $T_{\text {dwell}} < T_{\text {dwell}}^{\text {opt}}$. Another possibility is the presence of two types of noise: *t*-dependent and *t*-independent noise in the time domain *t*. The first type of noise originates from electrical sources, regarded as a constant background when averaged over a certain *t*-range, denoted as $I_\text {signal}^{(1)}(t) = \text {const}$. Conversely, the second type is characterized by unpredictable and spiky noise components, whose intensity and emerging probability are random against *t*, expressed as $I_\text {signal}^{(2)}(t) = \sum A_i \cdot \delta (t - t_i)$. Here we assume the spike noise is given by the delta function with an amplitude $A_i$ at a time $t_i$, where *i* represents the number of events having the spike noises ($i=1,2,3...$). Note that $I_\text {signal}^{(2)}(t)$ can be more problematic, especially for short $T_{\text {dwell}}$, as the counts are generally not rich in spin-resolved ARPES experiments. Since the shorter $T_{\text {dwell}}$ can lead to instability in the proper accumulation of spectral information, it is essential to ascertain $T_{\text {dwell}}^{\text {opt}}$ in each measurement technique, though this is beyond the scope of this work.

**Figure 3:**
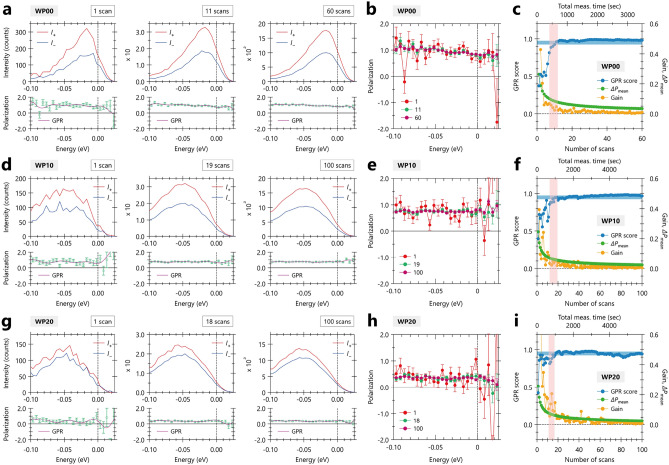
Evaluation of accuracy in spin-polarization extracted from the raw EDCs taken with different degrees of linear polarization as a function of accumulations in $$\text {Bi}_{2}$$$$\text {Te}_{3}$$ using Gaussian process regression (GPR) model. (**a**,**d**,**g**) Raw EDCs for positive (red) and negative magnetization (blue), along with the spin-polarization (green), for different numbers of scans, taken with different degrees of linear polarization achieved by changing angles of half-wavelength plate (WP), labelled as WP00, WP10, and WP20. Here, the first, critical ($$N_\text {cr}$$), and last number of scans ($$N_\text {emp}$$) are highlighted for $$N_\text {scan}$$’s. The definitions of $$N_\text {cr}$$ and $$N_\text {emp}$$ are given in the main text. (**b**,**e**,**f**) Comparison of spin-polarization, including uncertainty, between different number of scans with different angles of WPs. (**c**,**f**,**i**) GPR score (light blue) on the left axis, as well as gain and mean uncertainty of the spin-polarization $${\Delta }P_{\text {mean}}$$ (orange and green curves) on the right axis, as a function of numbers of scans, for different angles of WP. All the WP-dependent data were obtained through a random summation analysis, as described in Supplementary Note 1 and Fig. S1, where ten spectra are randomly selected from the raw spectra and summed up, virtually corresponding to a data acquisition with a dwell time of 1.0 sec.

**Figure 4:**
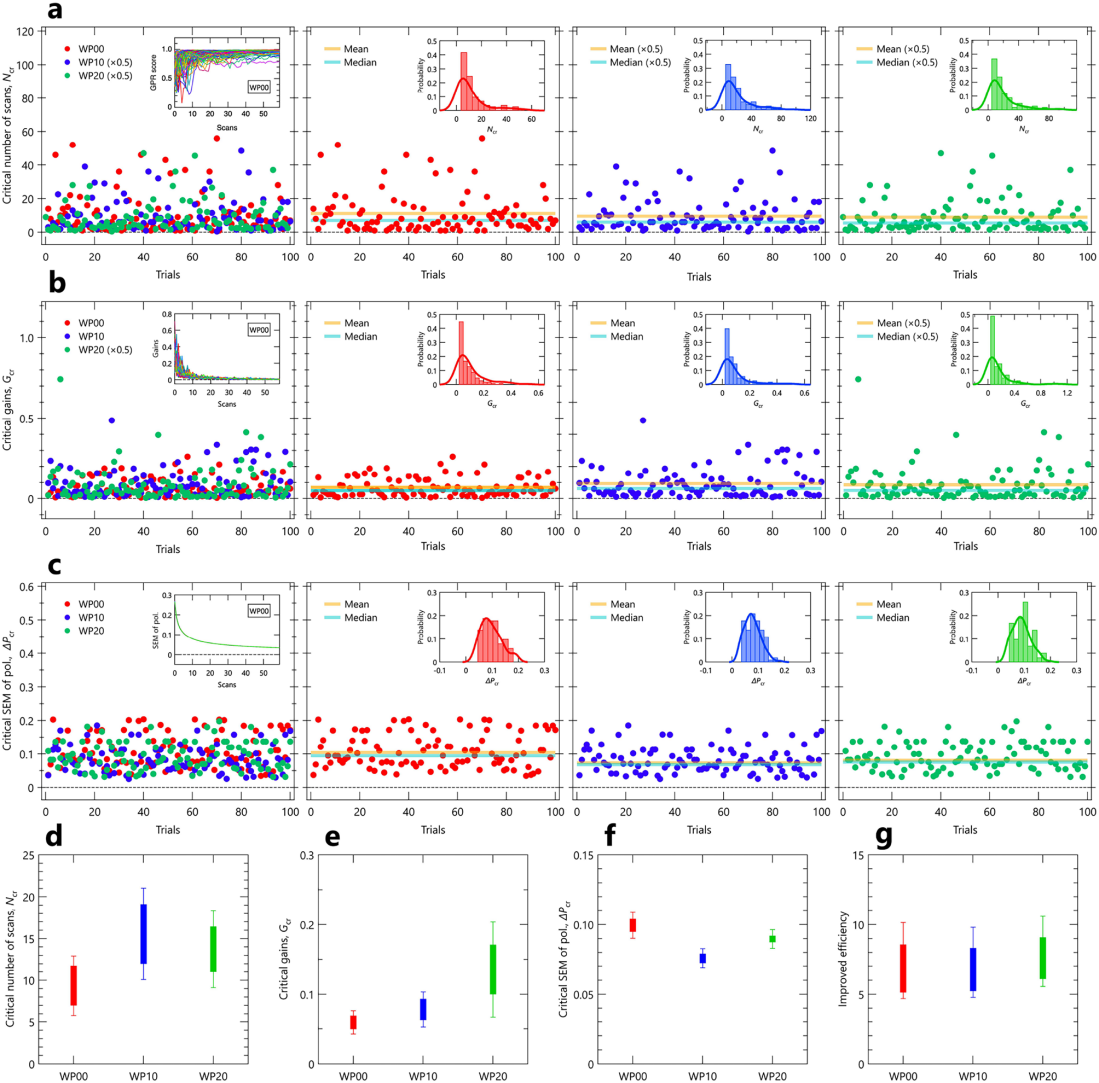
Evaluation of indicators obtained from the GPR model analysis as possible stopping criteria in spin-resolved ARPES experiments. (**a**) Scatter plot of the critical number of scans at which the GPR score exceeds 95$\%$, as a function of trials. A trial represents a sequential measurement accompanied by the multiple numbers of scan. The leftmost panel compares the critical numbers of scans $N_\text {cr}$ among different angles of half-wavelength plate, denoted as WP00, WP10, and WP20. The other panels display each critical number of scans, with the mean and median values indicated by the orange and sky blue lines. The inset of the leftmost panel shows the GPR scores as a function of numbers of scans, while the insets of other panels present the histogram of the critical number of scan. (**b**) and (**c**) Same as (**a**), except that the critical values of gains and mean uncertainty of the spin-polarization (${\Delta }P_{\text {mean}}$) at the 95$\%$ critical number of scan are displayed, respectively. (**d**−**f**) 95$\%$ critical number of scans, the critical values of gains and ${\Delta }P_{\text {mean}}$, ranging from the median to mean value with the standard error of the mean (SEM), for different WPs. (**g**) Efficiency for different WPs, obtained by $N_{\text {emp}}$/$N_{\text {cr}}$, where $N_{\text {emp}}$ represents the number of scans determined by following the empirical stopping criterion while $N_{\text {cr}}$ is the present 95$\%$ critical number of scan.

### $P_y$ dependence

In the previous section, we presented that the GPR model successfully predicted a reasonable stopping criterion for extracting the spin-polarization in $$\text {Bi}_2$$$$\text {Te}_3$$, where the electronic states are almost fully polarized. However, there exists a concern whether the present analysis is applicable to the dataset with lower spin-polarization, which is generally more difficult to probe. Here, we thus expand the present GPR model analysis on the spin-resolved EDCs of $$\text {Bi}_2$$$$\text {Te}_3$$ with the varied magnitude of the in-plane component of spin polarization $$P_{y}$$ by adjusting the degrees of linear polarization^37,38^ (see Methods).

Figure 3 shows the evaluation of accuracy of the spin-polarization in spin-resolved EDCs on $$\text {Bi}_2$$$$\text {Te}_3$$ as a function of $$N_\text {scan}$$. The data were taken with different degrees of linear polarization achieved by changing angles of the half-wavelength plate (WP), labelled as WP00, WP10, and WP20, resulting in different magnitudes of the in-plane component of spin polarization $$P_{y}$$^37,38^. It should be note that all the WP-dependent data were analyzed through a random summation analysis procedure, as described in Supplementary Note 1 and Fig. S1, to assess the robustness of the present GPR model analysis solely against the magnitude of $$P_y$$. In brief, we randomly selected ten spectra from the raw spectra and summed them up, effectively simulating a’ data acquisition with a dwell time of 1.0 sec. The virtual acquisition is repeated until satisfying the empirical stopping criterion of spin-resolved experiments. We further repeated a series of data acquisition and analysis by 100 times to suppress an instability due to the random data summation. In the following, we present the representative and overall results in Figs 3 and 4, respectively, where the representative results in Fig. 3 are from a sequence giving the mean value of the critical number of scan ($$\overline{N_\text {cr}}$$) from all the results in Fig. 4.

Figure 3a,d,g shows the spin-resolved EDCs for positive (red) and negative magnetization (blue), along with the spin-polarization (green) and the GPR prediction (purple), as a function of (virtual) scans, following the same manner used in Fig. 2. The enlarged view of the spin-polarization at different $N_\text {scan}$’s and the outcome of the GPR model analysis are presented in Fig. 3b,e,h and c,f,i, respectively. The magnitude of spin-polarization is varied from $\sim$100$\%$, $\sim$80$\%$, and $\sim$40$\%$ for WP00, WP10, and WP20, as shown in Fig. 3b,e,h. The overall trends regarding the accumulation of the *S*/*N* ratio of the spin-resolved EDCs and spin-polarization, as well as the results derived from the GPR model analysis, are essentially similar to what is observed in the previous section. As a results, the obtained $N_\text {cr}$ values are 11, 19, and 18 scans against the $N_\text {emp}$ values of 60, 100, and 100 scans, for WP00, WP10, and WP20, respectively. Accordingly, the improved efficiency of spin-resolved ARPES experiments can be estimated by $N_\text {emp} / N_\text {cr}$, as 5.5, 5.3, and 5.6 for WP00, WP10, and WP20, respectively, by reasonably reducing the acquisition number of scans.

Finally, we present the comprehensive results obtained through 100 repetitions of data acquisitions and analyses. Figure 4a shows critical number of scans $N_\text {cr}$, which is defined as the lowest number of scans giving the GPR score higher than 95$\%$, as a function of the number of trials $N_\text {tr}$. Similarly, Fig.  4b,c show the $N_\text {tr}$-dependence of the gains and mean of uncertainty of the spin polarization at $N_\text {cr}$, denoted as $G_\text {cr}$ and ${\Delta }{P}_\text {cr}$, respectively. In Fig.  4a–c, the leftmost panels compare all the results obtained by WP00, WP10, and WP20, where the inset panel shows the source data for determining the critical values. In right-side panels, each of results, obtained by WP00, WP10, and WP20, is individually shown from left to right, respectively, along with its mean and median values, as indicated by the orange and sky blue lines, respectively. In those insets, the $N_\text {tr}$-dependence of each of the values is shown by the histogram, along with the kernel density estimation (KDE), as indicated by the line. Note that some dataset includes a reduction by half to enhance visibility, while meaning that, in turn, such datasets have larger fluctuations. As easily seen in the leftmost panels of Fig.  4a–c and their insets, the data fluctuations are minimal for ${\Delta }{P}_\text {cr}$, compared with $N_\text {cr}$ and $G_\text {cr}$. At first glance, this observation might give an impression that ${\Delta }{P}_\text {cr}$ is the most robust and can be considered as a suitable stopping criteria in the spin-resolved ARPES experiments. However, such an interpretation warrants caution. Notably, the observed $P_y$ dependence on the data fluctuations of $N_\text {cr}$ and $G_\text {cr}$ seems reasonable, as the data fluctuations are expected to increase sequentially from the WP00, WP10, and WP20 results, corresponding to the decrease in $P_y$ or counts. This suggests that the observed data fluctuations likely stem, at least partially, from issues related to dwell time optimization $T_{\text {dwell}}^{\text {opt}}$ and/or noise, particularly becoming more pronounced for lower counts, as discussed in the previous section. Therefore, the perceived robustness of ${\Delta }{P}_\text {cr}$ may include artificial components, as improvements in ${\Delta }{P}_\text {cr}$ may occur even with greater noise compared to the signal ($I_\text {noise} > I_\text {signal}$). Hence, it is mandatory to assess the accumulation of spin polarization, not solely relying on ${\Delta }{P}_\text {cr}$ (or counts), but also considering the GPR score and gains derived from the GPR model analysis presented here. More importantly, despite the presence of data fluctuations, the GPR model analysis in this study provided an average 5-10 times improvement in efficiency (Fig.  4g), by utilizing the GPR score as the stopping criterion in spin-resolved ARPES experiments. We should point out that further applications and examinations using various types of spin-resolved ARPES datasets are necessary, to strengthen the reliability of the present analytical methods and identify the most suitable and automated stopping criteria, which we leave for future work. Despite this, our present work successfully demonstrates the promising possibility of designing efficient spin-resolved ARPES experiments utilizing measurement informatics.

## Summary

In summary, we investigated the application of GPR model to enhance the efficiency of spin-resolved ARPES experiments, using the representative topological insulator $$\text {Bi}_2$$$$\text {Te}_3$$ as a test material. The accumulation of spin polarization information was assessed through the analysis based on the GPR model. By utilizing the GPR score as the stopping criterion of spin-resolved ARPES experiments, a significant reduction in time costs was achieved by 5-10 times compared to empirical criteria. Our findings are thus emphasizing the promising importance of measurement informatics in advancing the field of spin-resolved ARPES experiments and other spectroscopic measurements.

## Methods

### Materials and experiments

The $$\text {Bi}_2$$$$\text {Te}_3$$ single crystals were fabricated by using the modified Bridgman method^39,40^. ARPES and spin-resolved ARPES experiments were performed at laser-based micro spin-resolved ARPES system ($$\mu $$-SARPES), developed at the Research Institute for Synchrotron Radiation Science, Hiroshima University using a hemispherical electron analyzer (Scienta-Omicron, DA30) equipped with two VLEED-type spin detectors. Detailed information about the $$\mu $$-SARPES system can be found in Ref.^36^. The data presented in this study were measured using a photon energy of 6.39 eV below 40 K with linear polarization in ultrahigh vacuum conditions better than $$1\times $$ $$10^{-9}$$ Pa. The flat and clean surface of the samples were obtained by cleaving *in situ* using the Scotch tape method at a pressure of $$5\times $$ $$10^{-7}$$ Pa at room temperature in a preparation chamber. While the vacuum level during cleaving resulted from connecting the vacuum to a load-lock chamber, the cleaved samples were then rapidly transferred to the measurement chamber within 1 minute. The degrees of linear polarization (*DoLP*) were controlled by the half-wavelength plate (WP) against the horizontally polarized incident light. Namely, we can continuously change the polarization direction from horizontal (*p*) to vertical (*s*) by varying $$0^\circ $$ to $$45^\circ $$ of the angle $$\theta $$, defined as the angle between the laser incident axis and the optical axis of the WP. We utilized three different $$\theta $$ angles ($$0^\circ $$, $$10^\circ $$, and $$20^\circ $$), yielding the different degrees of *p* and *s* polarizations ($$DoLP_p$$, $$DoLP_s$$) as (100$$\%$$, 0$$\%$$), (88.3$$\%$$, 11.7$$\%$$), and (58.7$$\%$$, 41.3$$\%$$), respectively. Note that we labeled these setups as WP00, WP10, and WP20, with respect to the $$\theta $$ angle providing fully *p*-polarized light. The energy and angular resolution were set to be approximately 5 meV (30 meV) and $$0.75^\circ $$, respectively, in the present ARPES (SARPES) experiments.

### Computations

To approximate spin-resolved ARPES spectra, we employed Gaussian process regression (GPR)^41^ using $\texttt {Python~3.7.16}$ and $\texttt {scikit-learn 1.0.1}$ package^42^ for GPR implementation. Gaussian process is a generalised nonlinear model capable approximating nonlinear spin-resolved ARPES spectral shapes in linear regression in feature space. In the present implementation, the observed spin-resolved ARPES spectrum $Y(X) = (y_1,..., y_n)$ is a function of the observed energy points $X = (x_1, ... ,x_n)$, where *n* is the number of energy points. We used the radial basis function (RBF) as a covariance function, which has been adopted for spectral measurements^26,27^. The RBF is defined by $R_{ij} = \exp (|x_i - x_j|^2 / 2l^2)$, where *l* is a length scale, a hyperparameter to be tuned. However, in the present implementation, we adopted fixed length scale $l = 0.05$ which exhibits the best performance based on manual tuning. Through GPR, a predicted mean $\mu$ and a standard deviation $\sigma$ are obtained. The goodness of fit of GPR is evaluated with the GPR score, i.e., the coefficient of determination $R^2 = 1 - u/v$, where the residual sum of squares $u = \sum (y - \mu )^2$ and the total sum of squares $v = \sum (y - {{\overline{y}}})^2$, respectively. Note that ${{\overline{y}}}$ is a mean of *y*. Details of the GPR is described in the literature^26,27,41^. To evaluate information gain of the measurement, we defined gain *G* as $G = \sum |\mu _{i-1} - \mu _{i}|/\sum \mu _{i}$, where $\mu _{i}$ is the predicted mean of GPR for the present scan and $\mu _{i-1}$ is that of the previous one. The execution time of a single GPR loop, for example, analyzing 600 datasets as shown in Fig.  2g, was approximately 1.6 sec on a machine with the following CPU specifications: Intel core i9, 2.4 GHz, 8 cores, which is adequately fast to be implemented in practical measurement procedures. We recommend using PC systems with equivalent performance for implementing the present computations in actual SARPES experimental systems.

## Supplementary Information

Supplementary Information.

## Data Availability

The datasets used and/or analysed during the current study are available from the corresponding author on reasonable request.
