# Supplementary material for: Efficiency improvement of spin-resolved ARPES experiments using Gaussian process regression
Source: Sci Rep. 2024 Sep 23;14:20970. doi: 10.1038/s41598-024-66704-8 (PMC11420225; doi:10.1038/s41598-024-66704-8)
Supplement: Supplementary file 1 — Supplementary Information. [file 41598_2024_66704_MOESM1_ESM.pdf]

Supplementary Information: Efficiency improvement of spin-resolved ARPES experiments using Gaussian process regression

Hideaki Iwasawa[1,2,3,*], Tetsuro Ueno[1,4,†], Takuma Iwata[5,6], Kenta Kuroda[5,6], Konstantin A. Kokh[7,8], Oleg E. Tereschenko[8,9,10], Koji Miyamoto[3], Akio Kimura[5,6], and Taichi Okuda[3,6]

[1]Synchrotron Radiation Research Center, National Institutes for Quantum Science and Technology, Sayo 679-5148, Japan

[2]NanoTerasu Center, National Institutes for Quantum Science and Technology, Sendai 980-8579, Japan

[3]Research Institute for Synchrotron Radiation Science, Hiroshima University, Higashi-Hiroshima 739-0046, Japan

[4]Quantum Materials and Applications Research Center, National Institutes for Quantum Science and Technology, Takasaki 980-8579, Japan

[5]Graduate School of Advanced Science and Engineering, Hiroshima University, Higashi-Hiroshima 739-8526, Japan

[6]International Institute for Sustainability with Knotted Chiral Meta Matter (WPI-SKCM^2), Hiroshima University, Higashi-Hiroshima 739-8526, Japan

[7]V. S. Sobolev Institute of Geology and Mineralogy, Siberian Branch, Russian Academy of Sciences, Novosibirsk 630090, Russia

[8]Saint Petersburg State University, 198504 Saint Petersburg, Russia

[9]Rzhanov Institute of Semiconductor Physics, Siberian Branch, Russian Academy of Sciences, Novosibirsk 630090, Russia

[10]Synchrotron Radiation Facility SKIF, Boreskov Institute of Catalysis, Siberian Branch, Russian Academy of Sciences, Kol'tsovo 630559, Russia

[*]iwasawa.hideaki@qst.go.jp

[†] ueno.tetsuro@qst.go.jp

Contents

Supplementary Note 1
Supplementary Figure S1

Supplementary Note 1: Random summation analysis

To effectively eliminate instability, possibly originating from a short dwell time, we performed a random summation analysis for Figs. 3 and 4. In this analysis, we randomly selected the number of spectra (N_{sum}) from the raw dataset of spin-resolved energy distribution curves (EDCs) measured with a dwell time (T_{raw_dwell}). We then summed the selected spectra up, resulting in 'a' summed spectrum, which effectively corresponds to a simulated spectrum regarded as a virtual scan with a dwell time of $T_{virt\_dwell} = T_{raw\_dwell} \times N_{sum}$. We iterated the number of scans (N_{virt_scan}) until satisfying an empirical stopping criterion (N_{emp}) of spin-resolved ARPES experiments. The N_{emp} is typically determined to satisfy $I_{\pm} > 10^4$, where I_+ and I_- represent the intensity of spin-resolved EDCs measured with positively and negatively magnetized targets, respectively. While the obtained dataset from this series of procedures, counted as one trial, enables performing the analysis utilizing Gaussian process regression (GPR), we executed multiple trials to suppress potential instability arising from the random selection of the spectra.

To assess the validity of this analysis, we applied the random selection analysis to the dataset taken with a short dwell time (T_{raw_dwell} = 0.1), as shown in Fig. S1, where we utilized different summation numbers (N_{sum} = 5 and 10, resulting in T_{virt_dwell} = 0.5 and 1.0, respectively) with 100 iterations of trials. The overall behaviors of the GPR score, gains, and the mean of the standard error of the spin-polarization are similar to those observed in the results obtained from the GPR model analysis on the raw dataset with different T_{raw_dwell} values of 0.5 and 1.0 sec., as shown in Fig. 2. The mean value of the critical number for N_{sum} = 5 and 10 is determined as N_{cr_mean} = 17.3 ± 1.7 and 11.1 ± 0.9, respectively, with the error represented by the mean of the standard error. These values are quantitatively consistent with those (18 and 9) obtained from the raw dataset with the dwell time of 0.5 and 1.0 sec., as demonstrated in Fig. 2. Accordingly, the improved efficiency of spin-resolved ARPES experiments (N_{emp}/N_{cr_mean}) is deduced as 6.9 and 5.4 for T_{virt_dwell} = 0.5 and 1.0, respectively, both comparable to the values of 6.7 and 6.7 for T_{raw_dwell} = 0.5 and 1.0. An advantage of this approach is that the simulation of the number of trials is possible, allowing for the statistical parameter evaluation. This capability is also useful for suppressing possible instability related to intensity fluctuations, which more or less inevitably exist in the practical measurements.

Supplementary Figure

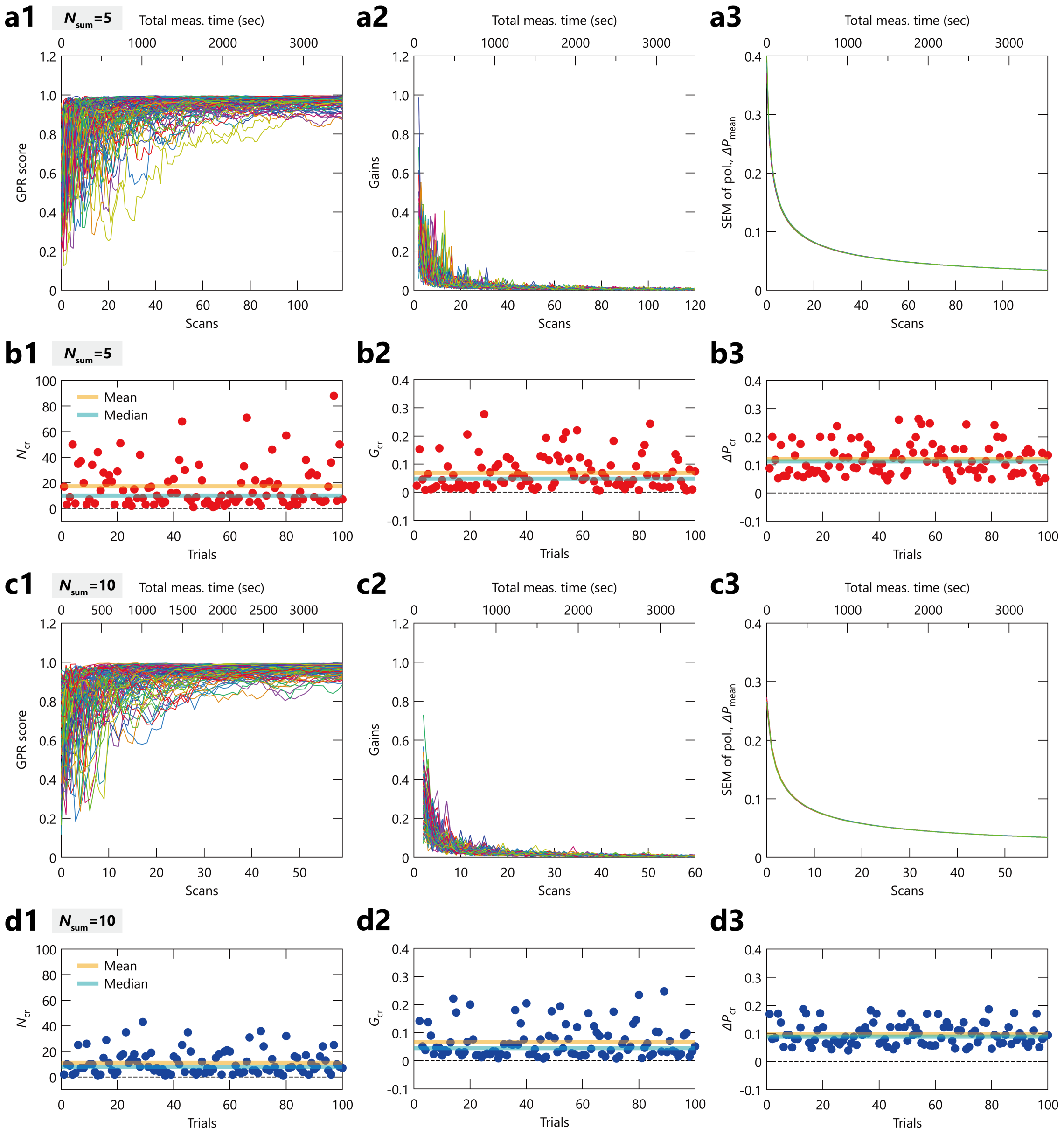

Fig. S1 Applications of random summation analysis. (**a1-a3**) and (**b1-b3**) Evaluation of various parameters obtained from the Gaussian process regression (GPR) model utilizing the random summation analysis for the summation number N_{sum} = 5: (**a1**) GPR score, (**a2**) gains (G), and (**a3**) mean uncertainty of the spin polarization ($ΔP_{mean}$), and (**b1**) the critical number of scans (N_{cr}), (**b2**) G_{cr} and (**b3**) $ΔP_{cr}$, defined as G and $ΔP_{mean}$ at N_{cr}, respectively. (**c1-c3**) and (**d1-d3**) Same as (**a1-a3**) and (**b1-b3**) except that they are obtained by N_{sum} = 10.
